# Red Blood Cells as Potential Repositories of MicroRNAs in the Circulatory System

**DOI:** 10.3389/fgene.2020.00442

**Published:** 2020-06-03

**Authors:** Liping Sun, Yang Yu, Beifang Niu, Deqing Wang

**Affiliations:** ^1^Department of Blood Transfusion, The First Medical Center, Chinese PLA General Hospital, Beijing, China; ^2^Computer Network Information Center, Chinese Academy of Sciences, Beijing, China; ^3^University of Chinese Academy of Sciences, Beijing, China

**Keywords:** erythrocytes, microRNA, erythropoiesis, extracellular vesicles, disease

## Abstract

The amount of erythrocyte-derived microRNAs (miRNAs) represents the majority of miRNAs expressed in whole blood. miR-451, miR-144, and miR-486, which are abundant in red blood cells (RBCs), are involved in the process of erythropoiesis and disease occurrence. Moreover, erythrocyte-derived miRNAs have been reported to be potential biomarkers of specific diseases. However, the function and underlying mechanisms of miRNAs derived from erythrocytes remain unclear. Based on a review of previously published literature, we discuss several possible pathways by which RBC miRNAs may function and propose that RBCs may serve as repositories of miRNAs in the circulatory system and participate in the regulation of gene expression mainly via the transfer of miRNAs from erythrocyte extracellular vesicles (EVs). In the whole blood, there are still other important cell types such as leukocytes and platelets harboring functional miRNAs, and hemolysis also exists, which limit the abundance of miRNAs as disease biomarkers, and thus, miRNA studies on RBCs may be impacted. In the future, the role of RBCs in the regulation of normal physiological functions of the body and the entire circulatory system under pathological states, if any, remains to be determined.

## Introduction

Research studies conducted in the past decades have shown that that microRNAs (miRNAs) play key roles in the process of erythropoiesis. Felli et al. (2005) first separated CD34^+^ hematopoietic progenitor cells from umbilical cord blood and found that the expression levels of miR-221 and miR-222 sharply decreased with the differentiation of CD34^+^ hematopoietic progenitor cells. Extensive evidence has shown that miR-451 and miR-144 are crucial to erythropoiesis and are located within the same gene cluster and regulated by the erythrocyte-specific transcription factor GATA1 (Fu et al., 2009; Papapetrou et al., 2010; Zhang L. et al., 2012; Xu et al., 2019). In addition, multiple miRNAs such as miR-200a and miR-150 have also been shown to be involved in the process of erythrocyte differentiation (Papapetrou et al., 2010; Zhang L. et al., 2012; Sun et al., 2015; Li et al., 2017). Interestingly, many miRNAs that are up- or downregulated during erythropoiesis may not be removed or degraded but selectively retained in mature red blood cells (RBCs) (Chen et al., 2008; Hamilton, 2010; Ryan and Atreya, 2011).

Only a few studies on the existence of miRNAs in mature RBCs have been performed to date due to limitations in methods for RNA testing (Lee et al., 1986; Rainen et al., 2002). In 2006, researchers for the first time detected small RNAs in RBCs (Rathjen et al., 2006). Chen et al. (2008) discovered that RBCs were the major contributors of miRNA expression in whole blood. In 2010, a report in the journal *Transfusion* showed that prolongation of the RBC preservation period alters the expression patterns of RBC-derived miRNAs (Kannan and Atreya, 2010). In 2012, a report in *Cell Host & Microbe* revealed that the upregulation of miR-451 in erythrocytes inhibits the reproduction of *Plasmodium falciparum* in patients with sickle cell anemia (Lamonte et al., 2012). With the development of high-throughput sequencing technologies, 287 known and 72 putative novel miRNAs were identified in erythrocytes (Doss et al., 2015), and multiple miRNAs (i.e., miR-451, miR-486-5p, and miR-144-3p) with high expression levels in whole blood were derived from RBCs (Wu et al., 2017). Recently, much attention has been paid to the role of miRNAs during RBC aging. miR-196a has been implicated in RBC storage-related damage *in vitro* (Sarachana et al., 2015). In addition, miR-142 has been shown to regulate RBC survival *in vivo* (Rivkin et al., 2017). However, the mechanism by which miRNAs function in RBCs remains unclear. Several observations provide important clues to elucidate the molecular mechanism of miRNAs in RBCs via extracellular vesicles (EVs) in research studies on *Plasmodium* infection (Regev-Rudzki et al., 2013; Mantel et al., 2016; Wang et al., 2017). Taken together, the above studies enumerate several important events involving RBC miRNAs.

This review discusses the potential roles of RBC miRNAs under physiological and pathological conditions, as well as possible mechanisms by which these function.

## Potential Roles of RBC miRNAs on Erythropoiesis Under Physiological Conditions *In Vivo*

Red blood cells contain an abundant and diverse array of miRNAs, with the similar levels to those in nucleated cells. MiRNAs in erythrocytes have a very unique expression pattern compared with human reticulocytes, which indicates that miRNAs are likely selectively retained and function in mature RBCs (Chen et al., 2008; Hamilton, 2010; Ryan and Atreya, 2011). MiRNAs derived from platelets, which are anucleate blood cells similar to RBCs, have been considered as disease biomarkers and play a critical role in the regulation of gene expression in physiological and pathophysiological conditions (Provost, 2017; Yao et al., 2019). Whether erythrocytes are in the above-mentioned situation as platelets remains unclear and thus is worth further investigation.

Researchers have identified 359 miRNAs in mature RBCs. The top 10 miRNAs with the highest expression levels in erythrocytes include miR-451a, miR-144-3p, miR-16, miR-92a, let-7, and miR-486-5p (Doss et al., 2015). Among these miRNAs, the expression level of miR-451 in erythrocytes is 10^4^ times higher than in granulocytes. miR-451 and miR-144 are closely clustered in the human and mouse genomes and promote erythroid differentiation (Masaki et al., 2007; Fu et al., 2009; Xu et al., 2019). In addition, miR-486 cooperates with GATA1 and GATA1s as an erythropoietic regulator (Shaham et al., 2015). Another study documented that miR-4732-3p within the erythroid-enriched miR-144/451 locus exhibits a high abundance in erythrocytes and targets two critical components (i.e., SMAD2 and SMAD4) of the transforming growth factor beta (TGF-β) pathway that is implicated in erythropoiesis, suggesting that miR-4732-3p plays an important role in promoting cell proliferation during erythroid differentiation (Choi et al., 2005; Masaki et al., 2007; Randrianarison-Huetz et al., 2010). Our team was the first to find that erythrocyte-derived miR-144-5p is affected by high-altitude hypoxic environments and may play a role in erythrocyte production (Sun et al., 2018). Based on the above analysis of miRNAs in RBCs, we proposed that a number of important miRNAs are involved in erythropoiesis.

Red blood cell-derived miRNAs show high expression levels, and some of them are delivered to recipient cells through vesicles and function (Mantel et al., 2016; Wang et al., 2017). It is thus essential to investigate whether the miRNAs related to erythroid differentiation in mature RBCs are transported from erythrocytes to hematopoietic progenitor cells and regulate their self-production under physiological conditions.

## Potential Roles of RBC miRNAs on Disease State and Diagnosis Under Pathological Conditions *In Vivo*

Investigations on RBC miRNAs in *Plasmodium* infection are the most extensive among RBC miRNA researches and have shown that erythrocyte-derived miRNAs (i.e., miR-451) participate in parasite pathogenesis, host defense, and the evolution of host polymorphisms driven by host interactions with these parasites (Rathjen et al., 2006; Lamonte et al., 2012; Taylor et al., 2013; Walzer and Chi, 2017).

Studies have shown that erythrocyte-derived miRNAs are associated with the abnormal state of RBCs and related diseases. Several investigations have documented various RBC miRNAs as biomarkers. For example, Duan et al. reported that miR-25-3p, miR-144-3p, and miR-486-5p in urinary sediments are mainly derived from urinary erythrocytes, which may be utilized as non-invasive candidate biomarkers for immunoglobulin A (IgA) nephropathy (Duan et al., 2016, 2017). Kira et al. showed that the expression levels of miR-3200-3p, miR-3200-5p, and miR-30b-5p derived from RBCs were downregulated in relapsing–remitting multiple sclerosis (Groen et al., 2018). miR-15a, miR-15b, and miR-499 have been demonstrated to be reduced in erythrocytes of prediabetic African-American adults (Fluitt et al., 2016). In addition, RBC miRNAs have been found to be linked to specific conditions. MiR-320 downregulation has been correlated with the upregulation of the CD71 protein (a key protein in the terminal differentiation process of reticulocytes) in erythrocytes from patients with sickle cell anemia (Chen et al., 2008). Our recent study showed that RBC miR-144-5p and miR-30b-5p are affected by high-altitude hypoxia (Sun et al., 2018). It has also been demonstrated that the expression level of miR-451 in RBCs is associated with the occurrence of chronic mountain sickness (Wang et al., 2015). Moreover, the upregulation of miR-486-5p, miR-92a, miR-16, and miR-451a in plasma are associated with increased hemolysis (Pritchard et al., 2012), which coincides with their high abundance in erythrocytes and in turn increases difficulty in using RBC miRNAs as biomarkers. Collectively, the role of erythrocyte miRNAs in disease incidence warrants investigation.

Previous studies have shown that the characteristics of erythrocytes are influenced by the tumor environment (Kuriki et al., 2007; de Castro et al., 2014). Pritchard et al. (2012) were among the first to highlight the problem of contamination of miRNAs derived from blood cells in the plasma of patients with cancer because of hemolysis occurring at the time of blood withdrawal or processing, thereby raising concern regarding their possible use as cancer biomarkers. A recent study has demonstrated that erythroblast-like Ter cells are induced in the spleen and promote tumor progression (Han et al., 2018), which suggests that erythroblasts may be influenced by the tumor microenvironment and undergo significant physiological changes. RBC miRNAs may be related to the occurrence of cancer and act as a trove of circulating biomarkers for cancer diagnostics. miRNAs are known to be involved in cancer (Pritchard et al., 2012), and miR-451, miR-144, miR-16, and miR-486, highly represented in erythrocytes, have been found deregulated in non-small cell lung cancer and acute leukemia (Abraham et al., 2017; Fang et al., 2018; Krakowsky et al., 2018; Liu et al., 2018). Leidinger et al. (2014) found that the expression profiles of cancer-specific miRNAs in whole blood samples are mainly determined by leukocytes. However, additional blood components, such as erythrocytes, platelets, or exosomes, are presumed contributing to the whole blood miRNome (Leidinger et al., 2014). Wu et al. (2017) analyzed the top blood-enriched miRNA markers (that were not expressed by colonic tissue) in the stool of colorectal cancer cases and controls, showing that an assay of erythrocyte-specific miRNA markers (i.e., miR-144-3p, miR-144-5p, miR-451a, miR-486-5p, miR-363-3p, and miR-20b-5p) can perform as fecal occult blood tests (FOBT), which measure the levels of heme in the stool. However, reports on the role of miRNAs encapsulated in RBCs in cancer are limited. There is a need to conduct studies to confirm the role of RBC-derived miRNAs in the tumor microenvironment.

Although multiple miRNAs derived from leukocytes and platelets in whole blood have been reported to be inextricably linked with the disease (Jeker and Bluestone, 2013; Esmailzadeh et al., 2017; Lam et al., 2018), RBCs as translationally inactive cells may present an abundant, diverse, and relatively stable picture of miRNA expression and have the potential in regulating the occurrence and development of diseases. Further related research should be thus conducted.

## Potential Roles of RBC miRNAs as Markers of Storage Lesions *In Vitro*

During storage, RBCs undergo a series of physiological and molecular changes. Previous studies have assessed the variations in miRNAs during storage of RBCs *in vitro* (Kannan and Atreya, 2010; Sarachana et al., 2015). Research has demonstrated that 4 of the 52 cell-apoptosis-related miRNAs (i.e., miR-96, miR-150, miR-196a, and miR-197) display a time-dependent increase in expression levels during a 20-day storage of RBCs (Kannan and Atreya, 2010). The miRNA profiles observed in stored RBCs may reflect storage-related lesions. miR-196a has also been implicated in RBC storage-related damage (Sarachana et al., 2015). Vu et al. (2017) demonstrated the existence of multiple intracellular Ago2-bound miRNAs in 24-h stored RBCs, highlighting that miRNAs in RBC may reflect its physiological state. Given the association of these miRNAs with RBC physiological status, it can be inferred that RBC miRNAs may be utilized as appropriate markers for RBC storage lesions. Currently, there is no definite “gold standard” for determining RBC quality, *in vivo* survival rate, and function after blood transfusion (Ryan and Atreya, 2011). Therefore, miRNAs might be potentially employed role in predicting safety and efficacy of blood products.

The above summarizes the possible roles of miRNAs derived from RBCs in physiological and pathological conditions (Table 1, the miRNAs indicated in italics are associated with several characteristics). However, research on the function of these large amounts of miRNAs in RBCs is limited. We have collected numerous reports and review the mechanisms of erythrocyte-derived miRNAs.

**TABLE 1:** Red blood cell-derived microRNAs (miRNAs) and their major characteristics.

**MiRNA**	**Major characteristics**	**References**
*MiR-451a*, *miR-144-3p*, *miR-16*, *miR-92a*, let-7, *miR-486-5p*	With the highest expression levels in erythrocytes	Doss et al., 2015; Wu et al., 2017
*MiR-451*, *miR-144*, miR-4732-3p, *miR-486*	Associated with erythropoiesis	Choi et al., 2005; Masaki et al., 2007; Fu et al., 2009; Randrianarison-Huetz et al., 2010; Doss et al., 2015; Shaham et al., 2015; Sun et al., 2018; Xu et al., 2019
*MiR-451a*, miR-140	Associated with malaria infection	Rathjen et al., 2006; Lamonte et al., 2012; Taylor et al., 2013; Walzer and Chi, 2017
MiR-320	Correlated with sickle cell anemia	Chen et al., 2008
*MiR-451*, *miR-16*, *miR-92a*, *miR-486-5p*	Associated with RBC hemolysis	Pritchard et al., 2012
MiR-25-3p, *miR-144-3p*, *miR-486-5p*	Non-invasive candidate biomarkers for IgA nephropathy	Duan et al., 2016, 2017
*MiR-144-5p*, *miR-30b-5p*	Correlated with high-altitude hypoxia	Sun et al., 2018
MiR-3200-3p, miR-3200-5p and *miR-30b-5p*	With different expression patterns in relapsing–remitting multiple sclerosis	Groen et al., 2018
MiR-15a, miR-15b, and miR-499	Associated with diabetes	Fluitt et al., 2016
*MiR-451*	Associated with the occurrence of chronic mountain sickness	Wang et al., 2015
*MiR-451a*, *miR-144-3p*, *miR-16*	Associated with cancer	Jeker and Bluestone, 2013; Leidinger et al., 2014; Abraham et al., 2017; Esmailzadeh et al., 2017; Fang et al., 2018; Krakowsky et al., 2018; Liu et al., 2018
*MiR-144-3p*, *miR-144-5p*, *miR-451a*, *miR-486-5p*, miR-363-3p, miR-20b-5p	Associated with colorectal cancer	Wu et al., 2017
MiR-96, miR-150, miR-196a and miR-197	Associated with storage lesions	Shaw et al., 2006; Kannan and Atreya, 2010; Ryan and Atreya, 2011; Sarachana et al., 2015

## MiRNAs May Target mRNA Sites in RBCs

Mature miRNAs bind to the 3′-untranslated region (UTR) sequence of target genes to form RNA-induced silencing complexes that influence protein synthesis by regulating gene transcription or degrading cytoplasmic messenger RNAs (mRNAs) (Djuranovic et al., 2012). Early studies might have missed low levels of mRNAs in RBCs, which means that miRNAs may have no targets in RBCs and thus have no function. However, current investigations have confirmed several different types of long RNAs in RBCs and have shown that the number of genes expressed in mature RBCs (∼8,092 genes) are far less than those of other nuclear blood cells such as peripheral blood mononuclear cells (∼15,743 genes) and erythroid progenitor cells (∼15,113 genes) (Azzouzi et al., 2015; Doss et al., 2015). This indicates that mature erythrocytes still have thousands of transcripts that may provide unique insights into erythroid biology. Many of the most highly expressed genes in the erythrocyte transcriptome encode proteins that are strongly associated with erythroid differentiation. For example, SLC25A37 encodes mitoferrin-1, an important iron introducer for the synthesis of heme and iron–sulfur clusters in erythroblast mitochondria (Shaw et al., 2006). BNIP3L mediates mitochondrial clearance during reticulocyte terminal differentiation (Zhang J. et al., 2012). FLT encodes the ferritin light chain, which is the main component of intracellular iron storage (Ponka et al., 1998). Erythrocyte membrane protein band 4.1 (EPB41), another highly expressed gene, which constitutes the cytoskeleton network of erythrocyte membranes, plays a key role in RBC shape and deformability (Conboy et al., 1986).

It is presumed that any residual translation in RBCs can be regulated by these miRNAs. As mentioned earlier in this review, researchers have studied the potential of miR-4732-3p in regulating Smad2 and Smad4 in RBCs, which show that miR-4732-3p overexpression inhibits SMAD4-mediated transcriptional activity (Chen et al., 2007; Dong et al., 2014). Although researches pointed out that there were no ribosomes in mature RBCs (Rifkind et al., 1964; Moras et al., 2017), we cannot exclude that some ribosomes could be neglected due to the electronic density of hemoglobin. A small amount of ribosomes may be enough to complete translation, in the case that mature RBCs retain a few specific RNA species. Further experiments are needed to verify whether miRNAs target mRNAs and function in erythroid biology within RBCs.

## RBC miRNAs Function as Ago2-Bound miRNA Complexes in the Circulatory System

The large circulating erythrocytic pool may be the source of miRNAs associated with protein (miRNPs), which are transported to recipient cells where these reprogram mRNA translation. Argonaute 2 (Ago2) binds and protects miRNAs from degradation in the circulatory system. As a basic functional unit, Ago2 directs the complex to specific targets on the regulated mRNA (Macfarlane and Murphy, 2010).

Previous studies have demonstrated the existence of multiple intracellular Ago2-bound miRNAs in RBCs that are stored for 24 h. Among these Ago2-bound miRNAs, miR-16-5p, miR-451a-5p, miR-486-5p, and miR-92a-3p are the most abundant miRNAs. Functional enrichment analysis of mRNA targets of major miRNAs identified molecules that are related to various diseases, biofunctions, and toxicity functions such as cardio-, hepato-, and nephrotoxicity (Vu et al., 2017). It has been reported that Ago2 complexes harbor a circulating miRNA population in human plasma (Zhao et al., 2016), and the Ago2–miRNA complexes produced by RBCs may be taken up by other cells, which in turn downregulate gene expression by targeting specific mRNAs (Arroyo et al., 2011; Zhang J. et al., 2012; Willeit et al., 2013; Vu et al., 2017).

Another study evaluated protein-bound miRNAs in RBC supernatants and showed that these induce monocyte suppression (Muszynski et al., 2015) which indicates that Ago-miRNAs derived from RBC, as a major protein–miRNA complex form, are likely transported to other recipient cells to exert their function. However, further research investigations are warranted.

## EV-Mediated miRNA Transmission May Be the Main Pathway by Which RBC miRNAs Function

Extracellular vesicles are the main miRNA carriers in the circulatory system (Koga et al., 2011; Benmoussa et al., 2016). Under normal physiological conditions, erythrocyte-derived EVs constitute 7.3% of EVs in whole blood, indicating that RBCs are one of the main sources of EVs in peripheral blood (Shah et al., 2008; Duan et al., 2016). EVs may contribute to the pathological process; for example, these may play an active role in atherosclerosis onset and progression (Li et al., 2016).

Juzenas et al. (2017) compared the miRNA content of RBCs with that of serum and exosomes and found a considerable overlap between all three fractions. Their results showed that 38 miRNA species are shared by serum, exosomes, and RBCs, suggesting that blood may contain exosomes derived from RBC and serum may contain miRNAs released by RBC (Juzenas et al., 2017). Researchers have detected that 78 miRNAs are present in exosomes of stored RBCs, and miR-125b-5p, miR-4454, and miR-451a are the most abundant miRNAs with potential functions (Huang et al., 2019). The above studies suggest the possibility that RBCs as repositories of miRNA function by releasing EVs that are loaded with miRNAs.

Several studies have shown that EV-mediated RNA transfer is an effective method for signal transduction that can modulate the biological processes of recipient cells (Huang et al., 2013; Thomou et al., 2017; Ying et al., 2017). Investigations have revealed that erythrocyte-derived EVs containing α-synuclein move across the blood–brain barrier through adsorptive-mediated transcytosis, which may be another mechanism for the initiation and progression of Parkinson’s disease (Matsumoto et al., 2017). Furthermore, exosome-like vesicles derived from RBCs are responsible for the transmission of pfPTP2 (a key protein of *P. falciparum*) between the donor cells and recipients, which is involved in the development of parasite infection (Regev-Rudzki et al., 2013), indicating that erythrocyte-derived EVs play key roles in cell-to-cell communication. Recent research has shown that EVs released from RBCs in large quantities during the blood phase of malarial infection are able to transfer human-derived Ago2–miRNAs (hAgo2–miR-451 and miR-140) complexes into the parasites within infected RBCs, which target the mRNAs of a critical parasite antigen *P. falciparum* erythrocyte membrane protein-1 (pfEMP1) and downregulated its expression (Wang et al., 2017). Mantel et al. (2016) revealed that infected RBC-derived EVs carrying hAgo2–miR-451 complexes are taken up by endothelial cells and alter vascular function during malaria infection. The abovementioned research provides important information on the function of miRNAs in RBCs via EVs, indicating that EV-mediated transmission may be the main functional pathway of RBC miRNAs.

**FIGURE 1:**
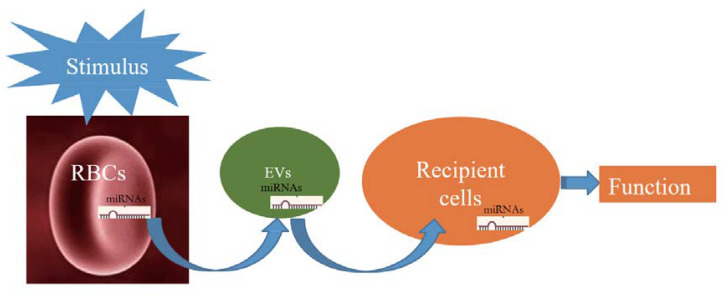
Schematic showing that when red blood cells (RBCs) are stimulated; they potentially release a large number of extracellular vesicles (EVs) that carry RBC-derived microRNAs (miRNAs) into recipient cells and function.

The pathway of miRNAs in erythrocytes acting through vesicles has only been reported in *Plasmodium*; however, its mechanism in erythrocytes remains unclear and thus warrants investigation. Figure 1 illustrates the possible transfer mechanism of erythrocyte miRNAs to the recipient cells via EVs, leading to functional changes. Although the mechanism of RBC-EV mediating miRNA function has not been extensively studied, RBC-EV is a member of EVs in the whole blood, which are widely reported to be absorbed by the recipient cells and play a role as a conventional mechanism of cell–cell communication (Thomou et al., 2017; Ying et al., 2017), suggesting that RBC-EVs have the potential of communicating with recipient cells in the tissues. Recent studies have shown that bone marrow, lungs, liver, spleen, and kidneys may be potentially used as targets of RBC miRNAs based on the following evidence as follows: (1) RBCs contain several important miRNAs that are involved in erythropoiesis (Masaki et al., 2007; Fu et al., 2009; Randrianarison-Huetz et al., 2010; Xu et al., 2019); (2) lungs are the main oxygen exchange organs where a large amount of RBCs accumulate, and RBCs may be one of the cell types of which miRNAs are influenced in lung cancer (Leidinger et al., 2014); and (3) RBC-EVs are enriched in the liver, spleen, and kidney (Matsumoto et al., 2017), and RBC miRNAs are possibly involved in kidney-related diseases (Abraham et al., 2017; Fang et al., 2018). These findings require further investigation.

## Discussion and Perspectives

In sum, miRNA expression patterns in whole blood are mainly influenced by erythrocytes, and RBCs may be significant repositories of miRNAs in the circulatory system, playing potential roles in erythropoiesis and disease development. MiRNAs derived from erythrocytes have been reported to be associated with disorders, meaning that RBC miRNAs may be used in the prediction and early detection of diseases, although the role and mechanisms of miRNAs in other cell types such as leukocytes and platelets in whole blood may weaken their application. Evidence from the studies cited herein suggests that the main mechanism by which RBC-derived miRNAs function may be that RBC miRNAs are carried in the EVs, transported to the recipient cells through the circulatory system, and ultimately affect mRNA expression in recipient cells. Based on existing reports, future research directions may focus on the role of erythrocyte-derived miR-144 and miR-451 on erythropoiesis in hematopoietic stem cells through EVs under physiological conditions. In addition, investigations on the potential of erythrocyte miRNAs as markers of disease diagnosis and disease state should be conducted.

In addition to the experimental verification on the function and mechanism of erythrocyte miRNAs, future studies should also clarify the possible causes of changes in erythrocyte miRNAs. According to the current literature, the reasons may be divided into two aspects: (1) mature RBCs may absorb exogenous miRNAs or release internal ones through the circulatory system (Mantel et al., 2016; Wang et al., 2017), and (2) different physiological or pathological conditions may affect the expression of miRNAs in erythroid differentiated cells, thereby affecting the residual amount of miRNAs in mature RBCs (Zhang L. et al., 2012; Shaham et al., 2015; Abraham et al., 2017). Additional studies on these aspects are warranted.

## Author Contributions

LS, DW, and BN composed the manuscript. LS and YY edited and revised the manuscript and the figure.

## Conflict of Interest

The authors declare that the research was conducted in the absence of any commercial or financial relationships that could be construed as a potential conflict of interest.
